# Amelioration of High-Insulin-Induced Skeletal Muscle Cell Insulin Resistance by Resveratrol Is Linked to Activation of AMPK and Restoration of GLUT4 Translocation

**DOI:** 10.3390/nu12040914

**Published:** 2020-03-27

**Authors:** Filip Vlavcheski, Danja J. Den Hartogh, Adria Giacca, Evangelia Tsiani

**Affiliations:** 1 Department of Health Sciences, Brock University, St. Catharines, ON L2S 3A1, Canada; fvlavcheski@brocku.ca (F.V.); dd11qv@brocku.ca (D.J.D.H.); 2 Centre for Bone and Muscle Health, Brock University, St. Catharines, ON L2S 3A1, Canada; 3 Department of Physiology, University of Toronto, Toronto, ON M5S 1A8, Canada; adria.giacca@utoronto.ca; 4 Department of Medicine, University of Toronto, Toronto, ON M5S 1A8, Canada; 5 Institute of Medical Sciences, University of Toronto, Toronto, ON M5S 1A8T, Canada; 6 Banting and Best Diabetes Centre, University of Toronto, Toronto, ON M5G 2C4, Canada

**Keywords:** resveratrol, high insulin, insulin resistance, IRS-1, mTOR, p70 S6K, AMPK, GLUT4

## Abstract

Insulin resistance, the hallmark of type 2 diabetes mellitus (T2DM), is linked to hyperinsulinemia, which develops to counterbalance initial peripheral hormone resistance. Studies indicate that chronically elevated levels of insulin lead to skeletal muscle insulin resistance by deregulating steps within the insulin signaling cascade. The polyphenol resveratrol (RSV) has been shown to have antidiabetic properties in vitro and in vivo. In the present study, we examined the effect of RSV on high insulin (HI)-induced insulin resistance in skeletal muscle cells in vitro and investigated the mechanisms involved. Parental and GLUT4myc-overexpressing L6 rat skeletal muscle cells were used. [^3^H]2-deoxyglucose (2DG) uptake was measured, and total and phosphorylated levels of specific proteins were examined by immunoblotting. Exposure of L6 cells to HI levels (100 nM) for 24 h decreased the acute-insulin-stimulated 2DG uptake, indicating insulin resistance. HI increased ser^307^ and ser^636/639^ phosphorylation of IRS-1 (to 184% ± 12% and 225% ± 28.9% of control, with *p* < 0.001 and *p* < 0.01, respectively) and increased the phosphorylation levels of mTOR (174% ± 6.7% of control, *p* < 0.01) and p70 S6K (228% ± 33.5% of control, *p* < 0.01). Treatment with RSV abolished these HI-induced responses. Furthermore, RSV increased the activation of AMPK and restored the insulin-mediated increase in plasma membrane GLUT4 glucose transporter levels. These data suggest that RSV has a potential to counteract the HI-induced muscle insulin resistance.

## 1. Introduction

Skeletal muscle is an important target tissue of insulin and is a homeostatic regulator of blood glucose levels. The increase in postprandial blood glucose levels results in insulin release by the β-cells of the pancreas into the bloodstream and delivery to the insulin target tissues, namely skeletal muscle, adipose and liver. Insulin increases uptake, utilization and storage of glucose by muscle and adipose tissue and inhibits endogenous glucose production by the liver [1]. Overall, the net effect of insulin is to return the postprandially elevated blood glucose levels back to physiological levels of around 5 mM. 

Muscle tissue responds to insulin and sequesters approximately 75–80% of the postprandially elevated blood glucose levels [2]. Insulin action in muscle is initiated by binding to its receptor, resulting in increase of the receptor tyrosine kinase activity and downstream tyrosine phosphorylation of insulin receptor substrate-1 (IRS-1), activation of phosphatidylinositol-3 kinase (PI3K) and protein kinase B/Akt, and GLUT4 glucose transporter translocation to plasma membrane allowing an increase in glucose uptake [1,3,4].

Muscle insulin resistance is a key contributor to impaired glucose tolerance and leads to type 2 diabetes mellitus (T2DM). Insulin resistance is strongly associated with nutrient overload (such as elevated levels of free fatty acids (FFA), glucose and/or amino acids [5,6]), cellular stress [7,8], inflammation [9,10] and high levels of circulating insulin, known as hyperinsulinemia [11]. 

Numerous studies have established that serine phosphorylation of IRS-1 leads to impaired insulin action and contributes towards insulin resistance [12,13,14,15,16]. Furthermore, serine kinases including protein kinase C (PKC) [17], glycogen synthase kinase 3 (GS3K) [18], c-Jun N-terminal kinase (JNK) [19,20,21], inhibitory kappa B (IκB) kinase (IKK) [13,22], mechanistic target of rapamycin (mTOR) [23,24,25] and ribosomal protein S6 kinase (p706SK) [26,27] have been shown to contribute to serine phosphorylation of IRS-1 [28] and insulin resistance. 

Adenosine monophosphate (AMP)-activated protein kinase (AMPK) is a cellular energy sensor that is activated upon increased AMP/ATP ratio and/or through phosphorylation by its upstream regulators including liver kinase B1 (LKB1), calmodulin-dependent protein kinase (CaMKKs) and transforming growth factor-β (TGF-β)-activated kinase 1 (TAK1) [29,30]. AMPK activation in muscle is shown under energy depletion states such as exposure to 2,4-dinitrophenol (DNP), an inhibitor of mitochondrial ATP production [31], and in response to exercise/contraction [32]. Compounds that are known activators of AMPK include metformin [33], salicylate [34], thiazolidinediones [35] and polyphenols such as naringenin, berberine, quercetin and resveratrol [36,37,38]. In recent years, AMPK has been viewed as an attractive treatment and/or prevention strategy against insulin resistance and T2DM [32,39,40].

Resveratrol (RSV), a polyphenol found in the skin of grapes and in red wine, has been demonstrated to activate AMPK and induce a two-fold increase in glucose uptake in L6 [37] and C2C12 skeletal muscle cells [41]. Treatment with RSV significantly improved glucose tolerance, insulin sensitivity, mitochondrial biogenesis and physical endurance in C57BL6/J mice fed a high-fat diet, while these effects were not seen in AMPKα1 or -α2 knockout mice, strongly indicating a key role of AMPK in mediating the effects of RSV [42]. Additionally, RSV has also been reported to prevent the fatty-acid-induced insulin resistance in L6 skeletal muscle cells [43].

According to the International Diabetes Federation (IDF), T2DM is a disease currently on the rise, with approximately 390 million people affected and approximately 4.5 million deaths reported in 2019 [44]. Most of the T2DM-related deaths are due to high incidence of diabetes complications due to lack of proper management/treatment of sustained hyperglycemia. Uncontrolled T2DM leads to liver, cardiovascular, kidney, eye, brain and nerve damage [45]. The current oral T2DM treatment strategy has limitations due to side-effects and reduced drug efficacy over time. For example, metformin, derived from French lilac, is widely used as a treatment for T2DM, however side-effects including gastrointestinal difficulties such as diarrhea, nausea, vomiting and cramps are often reported, while lactic acidosis remains a risk [46]. Other drugs, such as dipeptidyl peptide 4 (DDP-4) inhibitors or gliptins (sitagliptin, saxagliptin and linagliptin), used to treat T2DM have been associated with increased risk of heart failure, pancreatitis and even pancreatic cancer, although these associations have been disputed [47]. SGLT2 inhibitors have a favorable cardiovascular profile; however, they may favor ketoacidosis [48]. Therefore, there is an urgent need for new and more effective treatments, and the search of plant-derived compounds capable of counteracting insulin resistance is of high importance and can provide health benefits. As described in the discussion, initial studies with RSV in humans have given controversial results, but further investigation is required.

Initial and small defects in insulin signaling in skeletal muscle decrease postprandial glucose disposal. This leads to stimulation of insulin secretion by the pancreatic β-cell, resulting in a compensatory increase in insulin levels to maintain euglycemia. Eventually, the β-cell may fail, leading to hyperglycemia. The compensatory rise in insulin levels (hyperinsulinemia) acts to exacerbate insulin resistance, thereby contributing significantly to the pathogenesis of the disease [11]. Hyperinsulinemia contributes to impaired insulin signaling and metabolism in insulin target tissues. In vitro studies have shown that exposure to high insulin (HI) alone results in insulin resistance in C2C12 skeletal muscle cells [49]. Furthermore, a study found that exposure of 3T3-L1 rat adipocytes to HI resulted in IRS-1 degradation [50]. Moreover, treatment of primary mouse hepatocytes with HI abrogated the insulin-induced inhibition of glucose production [51]. Similarly, evidence from in vivo animal studies indicates that elevation of insulin levels by exogenous insulin administration results in insulin resistance [11,52,53]. Additionally, patients with primary insulinoma and no prior medical history of metabolic syndrome acquired insulin resistance, and complete resection of the insulinoma restored normal glucose metabolism and insulin sensitivity [54,55]. In obese individuals without T2DM, insulin hypersecretion and hyperinsulinemia are more prevalent than insulin resistance, indicating that hyperinsulinemia may precede and/or exacerbate insulin resistance [56,57].

Although studies clearly indicated attenuation of fatty-acid-induced insulin resistance by RSV, the effects of RSV on HI-induced insulin resistance in skeletal muscle cells have not been examined. The focus of the present study was to investigate the potential of RSV to counteract the HI-induced muscle insulin resistance. 

## 2. Materials and Methods 

### 2.1. Materials

Antibodies against IRS-1 (CAT# 2382, rabbit, 1:1000 dilution), phospho-ser307 IRS-1 (CAT# 2381, rabbit, 1:1000 dilution), phospho-ser636/639 IRS-1 (CAT# 2381, rabbit, 1:1000 dilution), Akt (CAT# 9271, rabbit, 1:1000 dilution), phospho-ser473 Akt (CAT# 9271, rabbit, 1:1000 dilution), phospho-thr308 Akt (CAT# 9275, rabbit, 1:1000 dilution), JNK (CAT# 9252, rabbit, 1:1000 dilution), phospho-thr183/tyr185 JNK (CAT# 9251, rabbit, 1:1000 dilution), mTOR (CAT# 9272, rabbit, 1:1000 dilution), phospho-ser2448 mTOR (CAT# 9271, rabbit, 1:1000 dilution), p70-S6K (CAT# 9202, rabbit, 1:1000 dilution), phospho-thr389 p70S6K (CAT# 9205, rabbit, 1:1000 dilution), AMPK (CAT# 2532, rabbit, 1:1000 dilution), phospho-thr172 AMPK (CAT# 2535, rabbit, 1:1000 dilution) and HRP-conjugated anti-rabbit secondary antibody (CAT# 7074, 1:2000 dilution) were from Cell Signaling Technology (Danvers, MA). Additionally, peroxidase-conjugated goat anti-rabbit IgG (CAT# 111-035-144, 1:1000 dilution) and c-myc antibodies (CAT# 3956, Sigma Life Sciences, 1:500 dilution) were purchased from Jackson ImmunoResearch Labs (West Grove, PA) and Sigma Life Sciences (St. Louis, MO). Polyvinylidene difluoride (PVDF) membrane, Luminol Enhancer reagents, molecular weight protein standards and electrophoresis reagents were purchased from BioRad. [^3^H]-2-deoxy-D-glucose was purchased from Perkin Elmer (Boston, MA). Resveratrol, cytochalasin B (CB) and BSA were purchased from Sigma (St. Louis, MO, USA). 

### 2.2. Cell Culture and Treatment

Parental and GLUT4myc-overexpressing L6 rat skeletal muscle cells were grown in α-MEM containing 5 mM glucose and 1% (v/v) antibiotic–antimycotic solution (100 U/mL penicillin, 100 µg/mL streptomycin and 250 ng/mL amphotericin B) in an incubator containing 5% CO_2_ at 37 °C as previously described [19]. Fully differentiated cells/myotubes were used in all experiments. The cells were incubated with or without 100 nM insulin, in the presence or absence of 25 µM RSV, or with RSV alone in 0% FBS-containing media for the time indicated in the figures. After the chronic exposure to high insulin, the cells were washed with acidic 0% FBS-containing α-MEM (pH 6.8) media for 5 min to dissociate insulin from its receptor, as described previously [58]. The response to acute insulin stimulation was examined by exposing the cells to 100 nM insulin after this wash. At the end of treatment, the cells were rinsed with HEPES-buffered saline (HBS) followed by a glucose transport assay or cell lysis and Western blotting. 

### 2.3. [^3^H]-2-deoxy-D-glucose (2DG) Uptake

To measure glucose uptake, the cells were exposed to HBS containing 10 μM [^3^H]2-deoxy-D-glucose for 10 min at room temperature, as previously reported [37]. Nonspecific uptake was assessed in the presence of 10 μM cytochalasin B. At the end of the 10 min exposure to the radioactive buffer and to stop the glucose transport through the plasma membrane, the cells were washed with ice-cold 0.9% NaCl, followed by cell lysis with 0.05 N NaOH. Cell-associated radioactivity was determined by liquid scintillation counting (PerkinElmer). Protein concentration was determined using the Bio-Rad assay. 

### 2.4. GLUT4myc Translocation Assay

GLUT4myc-overexpressing L6 myotubes were grown in 24-well plates; after treatment, the cells were washed with PBS and exposed to 3% paraformaldehyde (fixative) containing PBS for 10 min at 4 °C. The cells were then washed and incubated with 1% glycine containing PBS for 10 min at 4 °C and blocked using 10% goat serum containing-PBS for 15 min. The cells were incubated with the anti-myc antibody-containing blocking buffer (1:500) for 60 min at 4 °C, followed by washing with PBS and incubation with HRP-conjugated donkey anti-mouse IgG-containing blocking buffer (1:1000) for 45 min at 4 °C. The cells were rinsed with PBS, and O-phenylenediamine dihydrochloride (OPD) reagent was added for 30 min at room temperature. Finally, the reaction was stopped using 3 N HCl solution. The solution was collected, and the absorbance was measured at 492 nm. The OPD reagent is a substrate for HRP and produces a yellow-orange product measured at 492 nm by an enzyme-linked immunosorbent assay (ELISA) plate reader (Synergy HT, BioTek Instruments, USA). The changes in color intensity corresponds to the amount of GLUT4myc transporters present in the plasma membrane.

### 2.5. Western Blotting

At the end of the treatment, the cells were rinsed with HBS, followed by the addition of lysis buffer. The lysate was scraped off and solubilized in 3× SDS sample buffer. An equal amount of protein (15 μg) of each sample was separated by SDS-PAGE, transferred to a PVDF membrane and incubated for 1 h at room temperature with blocking buffer (1× TBS, 0.1% Tween-20 with 5% (w/v) nonfat dry milk), followed by incubation with the primary antibody overnight at 4 °C. The primary antibody was detected with a horseradish peroxidase (HRP)-conjugated anti-rabbit secondary antibody and ChemiGLOW reagent and visualized using FluroChem software (Thermo Fischer). The densitometry of the bands was measured using ImageJ software.

### 2.6. Statistical Analysis

The data are presented as the mean ± standard error (SE) of at least three to five separate experiments. Analysis of variance (ANOVA) followed by Tukey’s post hoc test was used to determine the significance of the differences between groups. Differences were considered statistically significant at *p* < 0.05. Calculations were performed using GraphPad software version 5.3.

## 3. Results

### 3.1. Resveratrol Restores the Insulin-Stimulated Glucose Uptake in High-Insulin-Treated Muscle Cells

**Figure 1:**
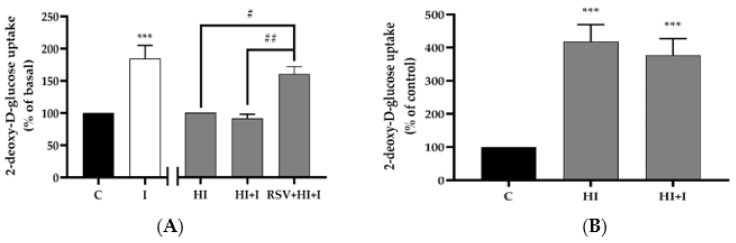
Effects of high insulin and resveratrol on insulin-stimulated glucose uptake. L6 myotubes were treated without (control, C) or with 100 nM insulin for 24 h (HI) in the absence or the presence of 25 μM resveratrol (RSV), followed by washing as indicated in the methods, acute stimulation with 100 nM insulin for 30 min (I) and glucose uptake measurement. The values are the mean ± SE of three to five independent experiments each performed in triplicate and expressed as percent of basal (**A**) or percent of control (**B**) (***p < 0.001 vs. control; #*p* < 0.05, ## *p* < 0.01 as indicated).

Acute stimulation of L6 myotubes with insulin (I; 100 nM, 30 min) resulted in a significant increase in glucose uptake (I: 184% ± 21% of basal control, *p* < 0.001; Figure 1A). Treatment with HI (100 nM, 24 h) significantly increased the basal glucose uptake (HI: 418.5% ± 51% of control, *p* < 0.001; Figure 1B). After the exposure to HI (100 nM, 24 h) the cells were washed with acidic (pH 6.8) 0% FBS-containing α-MEM media for 5 min to dissociate insulin from its receptor, followed by acute stimulation with insulin (100 nM, 30 min). Treatment of L6 myotubes with HI abolished the acute-insulin-stimulated glucose uptake (Figure 1A,B). Importantly, the presence of RSV (25 μM, 24 h) in HI-treated cells restored the acute-insulin-stimulated glucose uptake (HI: 100%, HI+I: 92% ± 6.0%, RSV+HI+I: 160% ± 11% of HI, *p* < 0.01; Figure 1A). These data indicate that the negative effect of HI on insulin-stimulated glucose uptake was prevented in the presence of RSV.

### 3.2. Resveratrol Prevents the High-Insulin-Induced Ser307 and Ser636/639 Phosphorylation of IRS-1 

**Figure 2:**
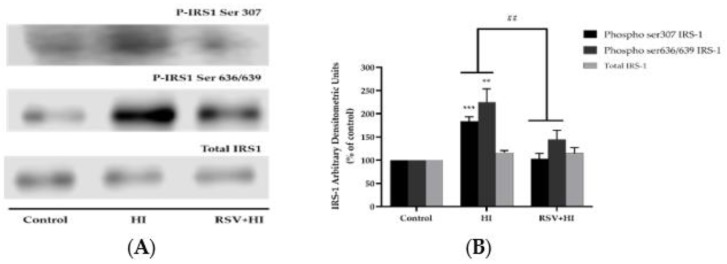
Effects of high insulin and resveratrol on phosphorylated IRS-1 ser^307^ and ser^636/639^ and IRS-1 expression. Whole-cell lysates from L6 myotubes treated without (control, C) or with 100 nM insulin for 24 h (HI) in the absence or the presence of 25 μM resveratrol (RSV) were prepared, resolved by sodium dodecyl sulfate polyacrylamide gel electrophoresis (SDS-PAGE) and immunoblotted for phosphorylated ser^307^ and ser^636/639^ or total IRS-1. A representative immunoblot is shown (**A**). The densitometry of the bands, expressed in arbitrary units, was measured using Scion software, and the data were presented as percent of control (**B**). The values are the mean ± SE of three to five separate experiments (***p < 0.001, **p < 0.01 vs. control; ##*p* < 0.01 vs. HI).

Previous studies performed in L6 muscle cells in vitro [59] and rat muscle tissue in vivo [14] have shown that increased serine (ser^307^ and ser^636/639^) phosphorylation of IRS-1 results in impairments in the insulin signaling cascade, leading to insulin resistance. Therefore, we investigated the effects of HI and RSV on serine phosphorylation and expression of IRS-1. Exposure of L6 myotubes to HI (100 nM, 24 h) resulted in a significant increase in ser^307^ and ser^636/639^ phosphorylation of IRS-1 (HI: 184% ± 9.3% and 225% ± 28.9% of control with *p* < 0.001 and *p* < 0.01, respectively; Figure 2A,B). Importantly, in the presence of RSV (25 μM), this phosphorylation of IRS-1 was blocked (RSV+HI: 103% ± 9.3% and 144% ± 19.6% of control, respectively; both *p* < 0.01). The total levels of IRS-1 were not significantly changed by any treatment (HI: 116% ± 4.5%, RSV+HI: 115% ± 12.3%; Figure 2A,B). 

### 3.3. Resveratrol Prevents the High-Insulin-Induced Phosphorylation/Activation of mTOR and p70 S6K

**Figure 3:**
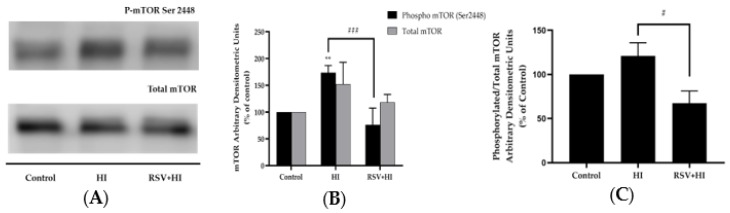
Effects of high insulin and resveratrol on mTOR phosphorylation and expression. Whole-cell lysates from L6 myotubes treated without (control, C) or with 100 nM insulin for 24 h (HI) in the absence or the presence of 25 μM resveratrol (RSV) were prepared, resolved by SDS-PAGE and immunoblotted for phosphorylated or total mTOR. A representative immunoblot is shown (**A**). The densitometry of the bands, expressed in arbitrary units, was measured using Scion software and expressed as percent of control (**B**). The data presented as the ratio of phosphorylated mTOR to total mTOR are shown (**C**). Values are the mean ± SE of three to four separate experiments (**p < 0.01 vs. control; #*p* < 0.05, ##*p* < 0.01 vs. HI).

Increased activation of muscle mTOR by nutrient overload [60,61] or exposure to high insulin [14] leads to increased serine phosphorylation of IRS-1, impaired insulin signaling and induction of insulin resistance. The effect of HI on muscle cell mTOR phosphorylation/activation was investigated next. Exposure of cells to HI resulted in a significant increase in mTOR phosphorylation (HI: 174% ± 6.7% of control, *p* < 0.01; Figure 3A,B), and this response was completely abolished in the presence of RSV (79% ± 15.9% of control, *p* < 0.001; Figure 3A,B). Although the total levels of mTOR showed a tendency to increase in response to HI exposure, statistical significance was not reached (HI: 153% ± 23.4%, Figure 3A,B). Treatment of the cells with HI in the presence of RSV did not affect total mTOR levels (RSV+HI: 118% ± 8.5% of control; Figure 3A,B). In addition, treatment of the cells with RSV alone did not affect phosphorylated or total mTOR levels (data not shown). The data expressed as the ratio of phosphorylated mTOR to total mTOR are shown in Figure 3C. The increase in the ratio of phosphorylated mTOR to total mTOR seen with HI was attenuated by RSV treatment (HI: 121% ± 15.0%, *p* < 0.05 compared to control, RSV+HI: 67% ± 14.0% of control, *p* < 0.05, compared to HI). 

**Figure 4:**
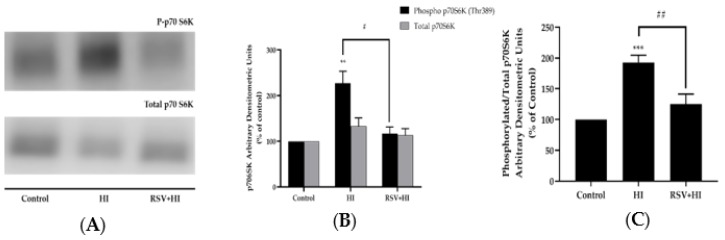
Effects of high insulin and resveratrol on p70 S6K phosphorylation and expression. Whole-cell lysates from L6 myotubes treated without (control, C) or with 100 nM insulin for 24 h (HI) in the absence or the presence of 25 μM resveratrol (RSV) were prepared, resolved by SDS-PAGE and immunoblotted for phosphorylated or total p70 6SK. A representative immunoblot is shown (**A**). The densitometry of the bands, expressed in arbitrary units, was measured using Scion software and expressed as percent of control (**B**). The data presented as the ratio of phosphorylated p70 6SK to total P70 S6K are shown (**C**). Values are the mean ± SE of four separate experiments (**p < 0.01, ***p < 0.001 vs. control; #*p* < 0.05, ##*p* < 0.01 vs. HI).

Additionally, p70 S6K, the downstream effector of mTOR, has been shown to cause serine phosphorylation of IRS-1, resulting in impaired insulin signaling and insulin resistance [26]. Exposure of cells to HI resulted in a significant increase in p70 S6K phosphorylation (HI: 228% ± 33.5% of control, *p* < 0.01, 4A, B). Importantly, the effect of HI was completely abolished in the presence of RSV (RSV+HI: 117% ± 19.0% of control, *p* < 0.05; Figure 4A,B). The total levels of p70 S6K were not significantly changed by any treatment (HI: 133% ± 17.9% of control, RSV+HI: 113% ± 14.7% of control; Figure 4A,B). In addition, treatment of the cells with RSV alone did not affect p70 S6K phosphorylation or expression (data not shown). The data expressed as the ratio of phosphorylated p70 S6K to total p70 S6K are shown in Figure 4C. The significant increase in phosphorylated p70 S6K to total p70 S6K seen with HI was attenuated by RSV treatment (HI: 193% ± 11.7% of control, RSV+HI: 125% ± 11.73% of control; *p* < 0.001 and *p* < 0.01, respectively).

### 3.4. Resveratrol Increased AMPK Phosphorylation in the Presence of HI

**Figure 5:**
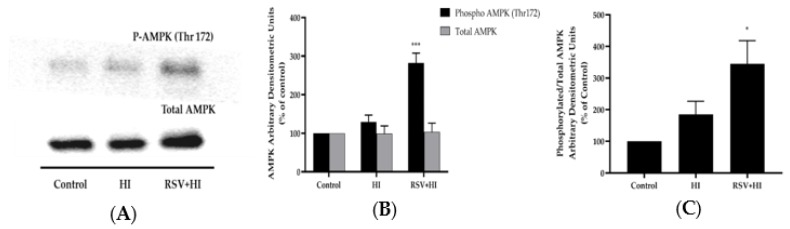
Effects of high insulin and resveratrol on AMPK phosphorylation and expression. Whole-cell lysates (15 µg) from L6 myotubes treated without (control, C) or with 100 nM insulin for 24 h (HI) in the absence or the presence of 25 μM resveratrol (RSV) were prepared, resolved by SDS-PAGE and immunoblotted for phosphorylated thr^172^ or total AMPK. A representative immunoblot is shown (**A**). The densitometry of the bands, expressed in arbitrary units, was measured using Scion software and expressed as percent of control (**B**). The data presented as the ratio of phosphorylated AMPK to total AMPK are shown (**C**). Values are the mean ± SE of four separate experiments (*p < 0.05, ***p < 0.001 vs. control).

Previous studies by our group and others have shown that RSV acutely (within 15 min to 2 h) phosphorylated/activated the energy sensor AMPK in L6 muscle cells [37]. In the present study, we investigated the effect of more prolonged (24 h) exposure to RSV on AMPK and the effect of RSV in conditions of HI. Treatment with RSV alone significantly increased the phosphorylation of AMPK (RSV: 213% ± 15% of control, *p* < 0.05; Figure 5A). Importantly, RSV increased the phosphorylation of AMPK in the presence of HI conditions (RSV+HI: 282% ± 25.6% of control, *p* < 0.001; Figure 5A,B). Treatment with HI alone did not significantly alter AMPK phosphorylation levels (HI: 129% ± 17.7% of control; Figure 5A,B). Furthermore, the total levels of AMPK were unchanged by any treatment (HI: 99.5% ± 19.9% of control, RSV+HI: 103% ± 23.0% of control; Figure 5A,B). RSV under HI conditions significantly increased the ratio of phosphorylated AMPK to total AMPK (RSV+HI: 345% ± 73.6% of control, *p* < 0.05).

### 3.5. Resveratrol Restores the Insulin-Stimulated GLUT4 Translocation in High-Insulin-Treated Muscle Ccells

**Figure 6:**
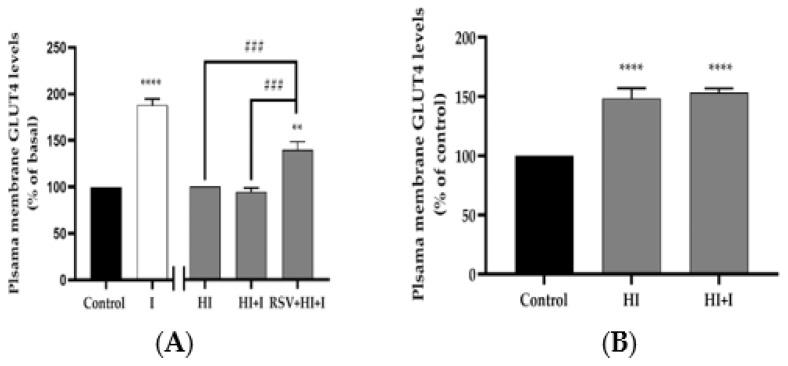
Effects of high insulin and resveratrol on GLUT4 translocation. GLUT4myc-overexpressing L6 myotubes were treated without (control, C) or with 100 nM insulin for 24 h (HI) in the absence or the presence of 25 μM resveratrol (RSV), followed by washing, as indicated in the methods, and acute stimulation with 100 nM insulin for 30 min (I). After treatment, plasma membrane GLUT4 glucose transporter levels were measured. Results are the mean ± SE of three independent experiments performed in triplicate, and expressed as percent of basal (**A**) or percent of control (**B**) (**p < 0.01, ****p < 0.0001 vs. control; ###*p* < 0.001 as indicated).

The increase in muscle glucose uptake with acute insulin stimulation is due to translocation of GLUT4 glucose transporter from an intracellular pool to the plasma membrane. To examine the effects of our treatment on GLUT4, we used L6 cells that overexpress a myc-labelled GLUT4 glucose transporter [62]. Acute stimulation of GLUT4myc-overexpressing L6 myotubes with insulin (100 nM, 30 min) resulted in a significant increase in GLUT4 plasma membrane levels (I: 188% ± 6.6% of basal control, *p* < 0.0001; Figure 6A). Chronic exposure of the cells to insulin (HI; 100 nM, 24 h), to mimic hyperinsulinemia, increased GLUT4 plasma membrane levels (HI: 148% ± 8.6% of control, *p* < 0.0001), (Figure 6B). HI abolished the acute-insulin-induced increase in GLUT4 plasma membrane levels, indicating insulin resistance (Figure 6A,B), and this response was restored in the presence of RSV (HI: 100%, HI+I: 92% ± 6.0 %, RSV+HI+I: 160% ± 11% of HI, *p* < 0.01; Figure 6A). 

## 4. Discussion

Increased plasma insulin levels (hyperinsulinemia) develop to counterbalance initial peripheral insulin resistance, but strong evidence indicate that this contributes to and exacerbates insulin resistance [11,55]. Patients with insulinoma that exhibit significantly elevated blood insulin levels have insulin resistance [63]. Mice transfected with extra copies of the insulin gene generating 2–4-fold increase in insulin levels exhibited hyperglycemia, hypertriglyceridemia and insulin resistance [64]. Furthermore, administration of insulin to rats to mimic hyperinsulinemia resulted in insulin resistance [53]. 

In the present study, we focused on examining the potential of RSV to prevent the HI-induced insulin resistance in muscle cells. The serine phosphorylation of IRS-1 has been shown to negatively impact insulin signaling by reducing the tyrosine phosphorylation of IRS-1 [12], association of phosphorylated tyrosine residues of IRS-1 with PI3K [15] and PI3K-Akt downstream signaling [12,14,23]. We found that treatment of L6 myotubes with HI for 24 h increased ser^307^ and ser^636/639^ phosphorylation of IRS-1. These data are similar to what was shown in CHO epithelial cells [50] and 3T3-L1 adipocytes [12]. Treatment of CHO epithelial cells, overexpressing rat IRS-1 or human insulin receptors and IRS-1, with insulin (100 nM) for 24 h significantly reduced the tyrosine phosphorylation while increasing serine phosphorylation of IRS-1, resulting in dose- and time-dependent degradation of IRS-1 [50]. Treatment of 3T3-L1 with insulin (100 nM) for 7 days resulted in significantly increased serine phosphorylation of IRS-1 [12]. Additionally, HI exposure resulted in IRS-1 degradation in 3T3-L1 adipocytes [50].

Our data indicate that treatment with RSV prevented the HI-induced serine phosphorylation of IRS-1, and in an attempt to understand the mechanisms involved we examined mTOR and p70 S6K. Studies have shown that increased phosphorylation/activation of mTOR and/or p70 S6K leads to increased serine phosphorylation of IRS-1 and the development of insulin resistance [12,14,23,26,60,65]. The studies by Pederson et al. [12] and Gual et al. [23] found that exposure of 3T3-L1 adipocytes to high insulin resulted in increased ser^307^ phosphorylation of IRS-1 and reduced PI3K and PBK/Akt activation [12,14,23], while treatment with the mTOR inhibitor rapamycin completely abolished these effects, indicating that mTOR phosphorylation/activation is involved in the phosphorylation of ser^307^ of IRS-1 [12,23]. Additionally, p70 S6K-deficient mice had lower ser^307^ and ser^636/639^ phosphorylation of IRS-1 and remained sensitive to insulin longer when fed a high-fat diet in comparison to their controls, suggesting that p70 S6K is involved in ser^307^ and ser^636/639^ phosphorylation of IRS-1 and the induction of insulin resistance [26]. A study by Ueno et al. showed that exposure of wild-type rats to chronic hyperinsulinemia markedly reduced the insulin-stimulated tyrosine phosphorylation of IR/IRS-1, association of IRS-1 with PI3K and phosphorylation/activation of Akt in muscle and liver tissue [14]. This impairment correlated with increased phosphorylation of mTOR, p70S6K and serine phosphorylation of IRS-1 [14]. Most importantly, subcutaneous injection of hyperinsulinemic Wistar rats with rapamycin (4 mg/kg b.w.) prevented the hyperinsulinemia-induced serine phosphorylation of IRS-1 and the reduction in the IR/IRS-1/PI3K/Akt signaling pathway in muscle and liver tissues [14]. In K/KAy mice with genetic obesity-associated insulin resistance that exhibit elevated phosphorylation levels of ser^307^ and ser^636/639^ of IRS-1, inhibition/deletion of raptor, a regulatory-associated protein of mTOR, resulted in improved glucose tolerance, suppressed ser^307^ and ser^636/639^ phosphorylation of IRS-1 and restored the insulin-induced PI3K activation and Akt phosphorylation in hepatic tissues [65]. Our study demonstrates that HI exposure increased muscle cell mTOR and p70 S6K phosphorylation/activation in association with increased ser^307^ and ser^636/639^ phosphorylation of IRS-1. These data are in agreement with the above-mentioned studies showing increased mTOR and/or p70 S6K activity and serine phosphorylation of IRS-1 [14,26,60]. Our data indicate a potential of RSV to abolish the HI-induced increase in mTOR, p70 S6K and IRS-1 (ser^307^ and ser^636/639^) phosphorylation.

In our study, both basal glucose uptake and plasma membrane GLUT4 levels were increased in the presence of HI, suggesting that the increase in basal glucose uptake is due to increased plasma membrane GLUT4 levels. Although there are currently no studies examining the effects of HI alone on GLUT4 plasma membrane levels, Huang et al. examined the effects of HI in combination with high glucose (HG) in L6 muscle cells [59]. Treatment of L6 myotubes with HI and HG increased the basal glucose uptake without affecting the GLUT4 plasma membrane levels but increased GLUT4 activity [59]. Furthermore, the combination of HI and HG resulted in reduced insulin-stimulated glucose uptake and GLUT4 plasma membrane levels [59]. Similarly to these data, we found that chronic exposure of L6 myotubes to HI significantly reduced the acute-insulin-stimulated glucose uptake (Figure 1) and plasma membrane GLUT4 levels (Figure 6). Importantly, in the presence of RSV, the HI-induced insulin resistance was prevented, and the insulin-stimulated glucose uptake and GLUT4 translocation was restored. These findings are the first to show that RSV can counteract the HI-induced insulin resistance. These effects of RSV are similar to the effects of metformin, shown to abrogate the HI-mediated reduction in insulin-stimulated glucose transport and plasma membrane GLUT4 levels [66]. 

Furthermore, we investigated the effects of HI and RSV on AMPK phosphorylation and expression. In a previous study by our group, stimulation of L6 muscle cells with RSV for 120 min resulted in robust phosphorylation/activation of AMPK and increased glucose uptake [37]. Treatment with compound C (CC), an AMPK inhibitor, abolished the effects of RSV, indicating that the effects of RSV are AMPK dependent [37]. Similarly, in C2C12 muscle cells, treatment with RSV (100 nM) for 60 min resulted in increased AMPK phosphorylation and glucose uptake that was attenuated by compound C [41]. Treatment of high-caloric diet-fed mice with RSV (22.4 ± 0.4 mg/kg/day) for 6 weeks significantly increased phosphorylation/activation of AMPK and improved insulin sensitivity [67]. In a study by Um et al., treatment of high fat diet fed C57BL6/J mice with RSV (400 mg/kg b.w./day) for 13 weeks significantly improved glucose tolerance, insulin sensitivity, mitochondrial biogenesis and physical endurance [42]. However, in high fat diet fed AMPKα1 or AMPKα2 knockout mice, RSV failed to elicit these effects, suggesting that the effects of RSV are AMPK mediated [42]. Studies have shown that the activation of AMPK reduces the activity of mTOR and p70 S6K [68,69,70]. In the present study, we found that under conditions of high insulin, RSV was able to significantly increase AMPK phosphorylation/activation. Our data suggest that the inhibition of mTOR and p70 S6K and the reduced serine phosphorylation of IRS-1 seen with RSV treatment under HI conditions (compared to HI alone) may be mediated by AMPK, and future studies utilizing an AMPK knockout approach should be performed to address this issue for clarification. 

There are a number of studies examining the effects of resveratrol administration in humans with T2DM and/or obesity. A study reported that oral administration of low dose RSV (5 mg twice a day for 4 weeks) in patients with T2DM resulted in improved insulin sensitivity, HbA1c, total cholesterol levels and oxidative stress [71]. In obese individuals, oral supplementation with RSV (150 mg/day for 30 days) resulted in improvement in glucose homeostasis and insulin sensitivity [72]. Furthermore, the metabolic profile and overall health was significantly improved by reducing adipose tissue lipolysis, hepatic steatosis, intrahepatic content, triglycerides content, inflammation markers (IL-6 and TNF-α) and, most importantly, increasing mitochondrial efficiency by activation of AMPK, PGC-1α and SIRT1 in muscle, thereby mimicking the effects of calorie restriction [72]. In another study in older overweight individuals with impaired glucose tolerance, administration of 1, 1.5 and 2 g of RSV per day for 4 weeks resulted in improved insulin sensitivity and postprandial glucose levels [73]. Moreover, administration of 500 g of trans-resveratrol three times a day for 90 days in 24 metabolic syndrome subjects resulted in decreased weight, fat mass, BMI index, waist circumference, area under the curve of insulin and total insulin secretion [74]. Similarly, another study showed that treatment of patients with T2DM with RSV (1 g/day for 45 days) reduced fasting plasma glucose, plasma insulin concentration and HbA1c, while decreasing homeostasis model of assessment of insulin resistance (HOMA-IR) and HOMA-β index [75]. Although the above studies have shown that RSV exhibits beneficial effects, others have shown little to no effect [76,77]. In well-controlled T2DM patients, oral supplementation of RSV (150 mg/day for 30 days) had no improvement in hepatic and peripheral insulin sensitivity or intrahepatic lipid content [76]. In metabolically impaired individuals, oral supplementation of RSV (1000 mg/day for 16 weeks) resulted in no changes in blood glucose and lipid homeostasis and no effects on body composition [77]. A study performed in obese individuals with no endocrine disorder showed that oral administration of higher dose of RSV (500 mg three times/day for 4 weeks) resulted in no improvement in insulin sensitivity, gene expression or inflammatory markers and no increase in AMPK and acetyl-CoA carboxylase activation [78]. Similarly, treatment with RSV (3000 mg/day daily for 8 weeks) in overweight or obese individuals with nonalcoholic fatty liver disease (NAFLD) but with no signs of endocrine disorder resulted in no improvement in NAFLD symptoms, including metabolic parameters and/or activation of molecular targets of RSV [79]. 

Overall, the majority of the studies have shown a beneficial effect of resveratrol administration in metabolically compromised humans, and more studies are required to establish the optimum dose and duration of treatment. There are several challenges and limitations in terms of past and current clinical studies involving RSV. In most studies, small sample sizes and single doses of RSV were used, resulting in improper understanding of the dose–response and sample size relationships. Moreover, long-term studies have never been performed and are required to examine the efficacy of RSV. Long-term studies are usually very expensive, and RSV is a naturally derived compound that is commercially available to everyone; therefore, the interest of the pharmaceutical industry to perform these trials is very low, and this might in part explain the lack of large-scale trials that would clearly elucidate the efficacy of RSV. 

## 5. Conclusions

**Figure 7:**
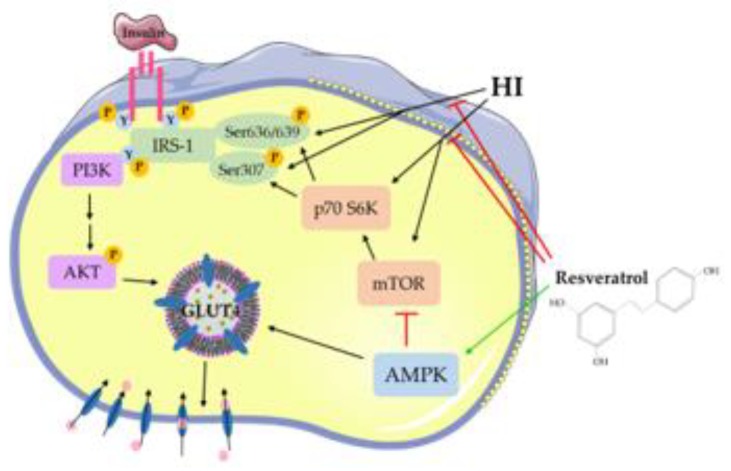
Resveratrol counteracted the high-insulin (HI)-induced muscle cell insulin resistance. RSV blocked the HI-induced serine phosphorylation of IRS-1 and phosphorylation/activation of mTOR and p70 S6K, while it enhanced the phosphorylation of AMPK.

The present study shows that exposure of L6 skeletal muscle cells to HI induces insulin resistance. Exposure to HI markedly increased the serine phosphorylation of IRS-1 and the phosphorylation/activation of mTOR and p70 S6K, while the acute-insulin-stimulated glucose uptake and plasma membrane GLUT4 glucose transporter levels were reduced. Importantly, RSV attenuated the HI-induced effects (Figure 7). RSV prevented the HI-induced reduction in insulin-stimulated glucose uptake and plasma membrane GLUT4 levels. Furthermore, RSV robustly increased the phosphorylation of AMPK (Figure 7). Our study is the first to show that RSV has the potential to counteract HI-induced muscle cell insulin resistance. Further studies are required to investigate the antidiabetic effect of RSV and, more importantly, to accurately elucidate the cellular mechanisms involved.
